# A call to update reproduction test criteria for *Enchytraeus crypticus*: why a 50% CV threshold no longer fits

**DOI:** 10.1007/s10646-025-03023-2

**Published:** 2026-01-14

**Authors:** Douglas Alexandre, Daniela Tomazelli, Luís Carlos Iuñes Oliveira Filho, Thiago Ramos Freitas, Rafaela Alves dos Santos Peron, Osmar Klauberg-Filho

**Affiliations:** 1 Santa Catarina State University, College of Agriculture and Veterinary, Department of Soils and Natural Resources, SC Lages, Brazil; 2 ECx Soil Health Solutions, SC Lages, Brazil; 3 Federal University of Santa Catarina (UFSC), Department of Environmental and Sanitary Engineering, SC Florianópolis, Brazil

**Keywords:** Enchytraeids, Ecotoxicological reproduction test, Validation criteria, Test validation

## Abstract

The ISO 16387 and OECD 220 guidelines provide standardized protocols for assessing the effects of contaminants on reproduction and survival in *Enchytraeus* species, key soil invertebrates involved in nutrient cycling and soil functioning. These guidelines include a validation criterion requiring the coefficient of variation (CV) for reproduction in control groups to not exceed 50%. This threshold was originally derived from tests using *Enchytraeus albidus*, a species with a longer test duration (42 days) and historically higher variability in reproductive outputs. However, the currently preferred species in ecotoxicological testing is *Enchytraeus crypticus*, which has a shorter test duration (varying between 21 and 28 days), higher reproductive consistency, and a markedly lower CV in empirical studies. Data compiled from 2006 to 2025, including recent studies across a range of artificial and natural soils, consistently show that *E. crypticus* control CVs remain below 30%, with many values between 6 and 15%. Rare outliers above 30% occurred under specific conditions. In contrast, other OECD and ISO guidelines for soil invertebrates (e.g., collembolans, earthworms, mites) already adopt stricter CV thresholds (≤ 30%), ensuring higher test sensitivity and comparability. We propose revising the CV acceptance threshold for *E. crypticus* reproduction tests to ≤ 30%, aligning validation criteria with demonstrated species performance and broader international standards. This adjustment would improve data precision, reduce false positives, and enhance reproducibility across laboratories, contributing to more robust Environmental Risk Assessments (ERAs) for plant protection products and other chemicals. We further call on the scientific community to contribute data and support harmonized efforts to refine guideline criteria for enchytraeids, strengthening their role in soil ecotoxicology and regulatory science.

## Introduction

Enchytraeids are small oligochaetes that play crucial roles in soil ecosystems, contributing to organic matter decomposition, nutrient cycling, and soil bioturbation (Didden 1993). Their close interaction with the porewater fraction of soil and multiple exposure routes (dermal, intestinal, and respiratory) make them particularly relevant for ecotoxicological studies (Castro-Ferreira et al. 2012; Lock and Janssen, 2001; Römbke 2003). These characteristics position enchytraeids as key organisms for assessing soil health and the environmental risks associated with pollutants. Given their ecological significance, ensuring precise and reliable testing protocols is critical not only for scientific accuracy but also for regulatory frameworks addressing environmental risk assessments (ERAs) and plant protection product (PPP) approvals.

To standardize such assessments, the ISO 16,387 (2023) and OECD 220 (2016a) guidelines outline test protocols for *Enchytraeus* species, using survival and reproduction as endpoints. While historically based on *Enchytraeus albidus* (Henle, 1837), a shift towards *Enchytraeus crypticus* (Westheide and Graefe 1992) has occurred due to its favorable traits for laboratory testing, including shortened generating time, higher reproductive consistency and broad environmental tolerance (Posthuma et al. 1997; Achazi et al. 1998; Peijnenburg et al. 1999; Kuperman et al., 2006 van Gestel et al. 2011; Westheide and Graefe 1992; ISO 2023a, b).

Despite this widespread adoption, the validation criteria, including the coefficient of variation (CV) threshold for reproduction (currently 50%), have not been updated to reflect the improved and consistent performance of *E. crypticus* under modern, standardized conditions. A critical discussion of this issue is presented in the following section, along with the rationale for revising the threshold to better suit the biological characteristics and empirical data associated with *E. crypticus.*

## Discussion

### Inadequacy of the current CV threshold

The current 50% CV threshold for enchytraeid reproduction was established from early ring tests with *E. albidus*, which were carried out under suboptimal and highly variable experimental conditions. As reported by Römbke and Moser (2002) and Weyers et al. (2002), these tests were affected by a lack of organism age synchronization, inconsistencies in culturing practices, and varying environmental conditions, as well as by the limited experience of some participating laboratories. The high variability observed in juvenile counts, approaching the 50% CV limit in many cases, was identified as a major challenge for data interpretation. These findings suggest that much of the variability attributed to *E. albidus* was methodological rather than intrinsic to the species.

Another contributing factor was the use of OECD artificial soil containing 10% sphagnum peat, a component known for its batch-dependent characteristics, which can introduce additional variability in toxicity tests. Recent discussions have emphasized the importance of substrate consistency (Van Hall and Van Gestel 2025). While no formal recommendation has been issued to reduce peat content, alternative artificial soil mixtures are increasingly considered. For instance, other OECD guidelines for soil invertebrates, such as collembolans and mites, recommend using only 5% peat (EPPO, 2003) to better simulate agricultural soils and reduce chemical sorption, thereby enhancing sensitivity.

Compared with *E. albidus*,* E. crypticus*, has shown more consistent *reproductive* performance across a wide range of soil types and test conditions (Kuperman et al., 2006; van Gestel et al. 2011). Empirical data consistently report CV values below 30% for *E. crypticus* reproduction, even in natural soils and under variable physicochemical conditions (see Table 1). This clear contrast highlights a misalignment between the current threshold and the performance characteristics of the most commonly used species.

**Table 1:** Reported CV values for *E. crypticus* reproduction in control groups

Study	Soil type	pH	Organic matter (%)	Clay (%)	CV (%)
Kuperman et al. (2006)	Artificial soil – OECD	6.0^a^	10	10	10.8
Kuperman et al. (2006)	Natural soil – SSL	5.2^a^	1.3	17	13
Castro-Ferreira et al. (2012)	Natural soil – LUFA 2.2	5.5^a^	4.0	6.0	11
Castro-Ferreira et al. (2012)	Natural soil – LUFA 2.2	5.5^a^	4.0	6.0	25
Castro-Ferreira et al. (2012)	Natural soil – LUFA 2.2	5.5^a^	4.0	6.0	18
Castro-Ferreira et al. (2012)	Natural soil – LUFA 2.2	5.5^a^	4.0	6.0	18
Castro-Ferreira et al. (2012)	Natural soil – LUFA 2.2	5.5^a^	4.0	6.0	17
Castro-Ferreira et al. (2012)	Natural soil – LUFA 2.2	5.5^a^	4.0	6.0	10
Castro-Ferreira et al. (2012)	Natural soil – LUFA 2.2	5.5^a^	4.0	6.0	25
Castro-Ferreira et al. (2012)	Natural soil – LUFA 2.2	5.5^a^	4.0	6.0	20
Castro-Ferreira et al. (2012)	Natural soil – LUFA 2.2	5.5^a^	4.0	6.0	17
Leitão et al. (2014)	Natural soil – Sandy loam	5.0^b^	5.74	23.5	5.8
Leitão et al. (2014)	Artificial soil – OECD	6.0^b^	10	n.a	36.9
Leitão et al. (2014)	Natural soil – Sandy loam	5.0^b^	5.74	23.5	10.8
Leitão et al. (2014)	Artificial soil – OECD	6.0^b^	10	n.a	11.6
Leitão et al. (2014)	Natural soil – Sandy loam	5.0^b^	5.74	23.5	39.5
Leitão et al. (2014)	Artificial soil – OECD	6.0^b^	10	n.a	8.3
Havelkova et al. (2014)	Artificial soil – OECD	6.0	10	20	6.0
De Menezes Oliveira et al. (2018)	Natural soil	6.4	15	48	20
Zortéa et al. (2018)	Artificial soil – TAS^c^	5.8^b^	5	20	16.3
Zortéa et al. (2018)	Natural soil – Oxisol	4.3^b^	3.9	55	12.3
Segat et al. (2020)	Artificial soil – TAS^c^	6.0	5	n.a	18.6
Ozturk et al. (2022)	Artificial soil – OECD	5.6	10	n.a	> 30
Van Hall and Van Gestel (2025)	Artificial soil – OECD	6.73^b^	10	n.a	16.7
Van Hall and Van Gestel (2025)	Artificial soil – OECD	6.71^b^	5	n.a	10.2
Van Hall and Van Gestel (2025)	Artificial soil – OECD	6.61^b^	2.5	n.a	15.3
Van Hall and Van Gestel (2025)	Natural soil – LUFA 2.2	5.39^b^	4.26	n.a	24.5
Van Hall and Van Gestel (2025)	Artificial soil – OECD	6.73^b^	10	n.a	7.2
Van Hall and Van Gestel (2025)	Artificial soil – OECD	6.71^b^	5	n.a	10.2
Van Hall and Van Gestel (2025)	Artificial soil – OECD	6.61^b^	2.5	n.a	8.7
Van Hall and Van Gestel (2025)	Natural soil – LUFA 2.2	5.39^b^	4.26	10.8	15.0
Van Hall and Van Gestel (2025)	Artificial soil – OECD	6.73^b^	10	n.a	11.6
Van Hall and Van Gestel (2025)	Artificial soil – OECD	6.71^b^	5	n.a	17.0
Van Hall and Van Gestel (2025)	Artificial soil – OECD	6.61^b^	2.5	n.a	8.6
Van Hall and Van Gestel (2025)	Natural soil – LUFA 2.2	5.39^b^	4.26	10.8	17.6
Van Hall and Van Gestel (2025)	Artificial soil – OECD	6.73^b^	10	n.a	47.7
Van Hall and Van Gestel (2025)	Artificial soil – OECD	6.71^b^	5	n.a	31.6
Van Hall and Van Gestel (2025)	Artificial soil – OECD	6.61^b^	2.5	n.a	6.4
Van Hall and Van Gestel (2025)	Natural soil – LUFA 2.2	5.39^b^	4.26	10.8	16.0
Van Hall and Van Gestel (2025)	Artificial soil – OECD	6.73^b^	10	n.a	15.6
Van Hall and Van Gestel (2025)	Artificial soil – OECD	6.71^b^	5	n.a	6.8
Van Hall and Van Gestel (2025)	Artificial soil – OECD	6.61^b^	2.5	n.a	12.5
Van Hall and Van Gestel (2025)	Natural soil – LUFA 2.2	5.39^b^	4.26	10.8	13.5

^a^ pH CaCl_2_^; b^ pH KCl; ^c^ Tropical artificial soil; n.a = not available

Maintaining the 50% threshold for *E. crypticus* undermines the precision, potentially masking subtle toxic effects and reducing the reliability of test results. Moreover, the leniency of this criterion contrasts sharply with stricter thresholds (≤ 30%) applied to other soil organisms, such as collembolans, earthworms, and mites (OECD 226, 2016d; OECD 222, 2016b; OCED 232, 2016c; ISO 11267, 2023b; ISO 11268-2, 2023; ISO 21285, 2019).

The coefficient of variation (CV), defined as the ratio between the standard deviation and the mean (CV = [SD/Mean] × 100), is a standard statistical metric used to assess the relative dispersion of data (Brian 1998) and is universally applied across ecotoxicological testing. Rather than revisiting the mathematical definition of CV, improving the robustness of CV values in test outcomes depends on minimizing sources of variability, both biological and procedural, that contribute to the standard deviation.

Our argument, therefore, centers on updating the threshold to reflect the reproducibility now achievable under current standardized conditions using *E. crypticus*. This adjustment would enhance the robustness of regulatory decision-making and improve the overall scientific integrity of ERA protocols.

### Evidence supporting threshold reduction

Table 1 compiles CV data from studies reporting reproduction results in control of *E. crypticus*. Although not exhaustive, this dataset highlights the consistently low variability observed across both artificial and natural soils. Importantly, only studies that explicitly report CV values in the body of the manuscript were included.

In artificial soils, CVs frequently remain well below 20%, with several studies reporting values as low as 6.0% (Havelkova et al. 2014), 6.4% (Van Hall and Van Gestel 2025), 8.3% (Leitão et al. 2014) and 10.8% (Kuperman et al., 2006), even under variable soil formulations, including tropical artificial soil (TAS) and reduced organic matter contents, underscoring the reproducibility of results under different artificial substrates (Leitão et al. 2014; Zortéa et al., 2018; Segat et al. 2020).

In natural soils, CVs similarly demonstrate low dispersion, frequently remaining under 25%. For example, *E. crypticus* achieved CVs as low as 5.8% (Leitão et al. 2014) and 12.3% (Zortéa et al., 2018) in field soils with highly contrasting properties, including pH (4.3–6.4), organic matter (1.3–15%), and clay contents (6–55%).

Outliers above 30%, such as values of 36.9%, 39.5% (Leitão et al. 2014), and a single instance of 47.7% (Van Hall and Van Gestel 2025), were rare and occurred under specific experimental or substrate conditions, often with higher peat content or unreported procedural details. These isolated cases do not represent the overall trend, which clearly supports a reproducibility ceiling around or below 30%.

Overall, the body of evidence reinforces the technical feasibility and ecological relevance of adopting a ≤ 30% CV threshold for *E. crypticus* reproduction tests. This adjustment would align its validation criteria with those already applied to other key soil invertebrates, including collembolans (*Folsomia candida*), earthworms (*Eisenia fetida*, *E. andrei*), and mites (*Hypoaspis aculeifer*), which are all subject to CV thresholds ≤ 30% (OECD 226, 2016d; OECD 222, 2016b; ISO 21285, 2019 OECD 232, 2016c; ISO 11267, 2023b; ISO 11268-2, 2023; ISO 21285, 2019). Such alignment would promote consistency across *taxa*, improve test sensitivity, and enhance confidence in comparative ecotoxicological assessments.

### Challenges and counterarguments

Reducing the CV threshold for *E. crypticus* to 30% presents clear scientific and methodological benefits, yet several operational challenges must be addressed to ensure feasibility and acceptance. These challenges encompass laboratory variability, resource disparities, financial constraints, and potential implications for historical datasets.

Laboratory variability stems from differences in expertise, infrastructure, and environmental controls across facilities. Laboratories with limited experience in handling *E. crypticus* may face challenges in meeting stricter thresholds due to inconsistencies in culturing practices, soil preparation, and environmental monitoring. These discrepancies can lead to variability in reproductive outcomes. To address this, investments in training, procedural adjustments, and equipment upgrades may be required, imposing short-term financial and logistical burdens. However, the long-term benefits of improved data quality outweigh these costs by enhancing the reliability and precision ERAs.

Historical datasets generated under the 50% criterion could be compromised by retroactive application. A phased implementation with statistical segregation of data by CV threshold can preserve historical utility while encouraging gradual compliance.

Advancements in laboratory practices have already reduced variability in *E. crypticus* tests. Standardized cultivation methods, accumulated expertise, and adherence to strict protocols have significantly improved test reliability. Routine reference tests with substances like carbendazim and boric acid, as recommended by ISO and OECD guidelines, further enhance sensitivity and consistency. Additionally, standardizing test organism size at the experiment’s outsets successfully applied to other species like those in fragmentation studies (e.g., Bandow et al. 2013) could minimize variability and improve test consistency for *E. crypticus*.

To facilitate the adoption of a 30% CV threshold, the following strategies are recommended:

- *Phased implementation*: Gradually introduce the 30% CV threshold to allow laboratories time to adapt their methods while maintaining compliance with existing standards.
- *Guidelines for standardization*: While existing efforts such as those by Castro-Ferreira et al. (2012) have contributed valuable guidance for *E. crypticus* testing, there remains a need to update and harmonize these protocols across laboratories. Emphasis should be placed on culturing practices, soil preparation, and environmental controls, particularly in the context of reducing interlaboratory variability and supporting regulatory acceptance.
- *Capacity building*: Promote training programs, inter-laboratory studies, and collaborative networks to enhance expertise. Additionally, support for improving laboratory infrastructure including access to more precise and standardized equipment is essential for achieving higher reproducibility and reducing experimental variability.
- *Distinguishing historical data*: Leverage statistical analyses and detailed documentation to categorize datasets by applied CV thresholds, preserving historical data’s relevance for regulatory and research purposes. In this sense, it is important that studies present CV values and not just indicate that the validity criterion was achieved.

By addressing these challenges through structured solutions, the transition to a more stringent CV threshold can improve the reliability and ecological relevance of *E. crypticus* testing while fostering broader acceptance across the scientific and regulatory communities.

### Implications for environmental risk assessments (ERAs)

Although the *E. crypticus* reproduction test is not mandatory for ERA in Europe and is considered an optional tool, since earthworm reproduction test (e.g., *E. fetida* or *E. andrei*) is the preferred standard, it is increasingly adopted in scientific studies where smaller-bodied, porewater-sensitive soil organisms provide complementary or more sensitive endpoints. This is particularly relevant for research on emerging contaminants, soil health, and within European regulatory frameworks that recognize multiple lines of evidence in ERA (Gregor et al. 2025).

In this context, lowering the CV threshold to 30% would also enhance test precision, allow the detection of more subtle treatment effects and reduce the likelihood of false positives. This adjustment strengthens the scientific robustness of *E. crypticus* data and increases confidence in their application to regulatory, comparative, and screening purposes. Furthermore, adopting a stricter CV threshold reinforces the ecological validity of the evaluations, as more precise and consistent data improves the quality of regulatory decision-making. This ensures that authorizations or restrictions for PPP and other chemicals are based on robust evidence regarding environmental impacts.

Therefore, we argue that rather than acting as a barrier, the implementation of a revised CV threshold should be viewed as a benchmark for data quality: one that is achievable through standardized methodologies and increasing laboratory capacity. As laboratories refine their protocols to meet this criterion, the overall reproducibility and scientific rigor of ecotoxicological data will improve, benefiting both regulatory frameworks and research efforts. Considering this we propose revising the validation criterion for *E. crypticus* reproduction tests to set the CV threshold for control groups at ≤ 30%, replacing the current 50% threshold. This update is justified by consistent empirical data and would align with validation standards for other invertebrate taxa. The change would enhance reproducibility, regulatory acceptance, and the ecological validity of *E. crypticus* as a model organism in soil ecotoxicology.

## Proposed appropriate analysis

To support the revision of validation criteria for *E. crypticus* reproduction tests, we propose a comprehensive, community-driven statistical analysis aimed at quantifying control variability and identifying its main sources. This initiative will integrate both published data and voluntarily submitted laboratory datasets, covering a wide range of experimental conditions.

Published data will be compiled through a time-limited systematic literature search (e.g. focussing on studies from 2006 onwards) using predefined inclusion and exclusion criteria. As a minimum, studies will be required to report control reproduction (mean), a measure of dispersion (e.g. SD, SE or CV), the number of replicates, and a basic description of test conditions (e.g. soil type, test duration, and species identity).

Voluntarily submitted datasets will complement this body of evidence and help to reduce publication bias, but the analysis will not depend exclusively on such submissions. The analysis will consider a broad set of environmental, biological, and procedural variables, including soil type, pH, organic matter content, age synchronization, culturing methods, test duration, and population origin, as well as a coded laboratory identifier. Data will be stratified by these parameters to assess their influence on CV outcomes. Additionally, simulation models will be applied to estimate the potential impact of adopting a stricter 30% CV threshold under different conditions.

To facilitate data collection, a dedicated online submission portal will be developed, offering standardized forms for uploading control datasets together with essential metadata. Laboratory and population identifiers will be stored in coded form during data processing so that the analysts can distinguish populations and institutions where relevant, while public summaries of the data will be anonymized. To minimize artefacts due to inconsistent naming (e.g. different ways of writing the same institution), the portal will use controlled vocabularies and drop-down menus for key fields such as institution, location and soil type, complemented by automated checks for obvious typographical variants.

This initiative is intended to generate transparent, evidence-based support for refining CV thresholds specific to *E. crypticus*, improve inter-laboratory reproducibility, and strengthen the ecological and regulatory relevance of standardized testing protocols. Furthermore, it serves as a platform to engage the scientific community in the collaborative development of species-specific validation criteria for future updates to ISO 16,387 and OECD 220.

## Data Availability

The data are available upon request.
